# Value of Cerebroplacental Ratio and Uterine Artery Doppler as Predictors of Adverse Perinatal Outcome in Very Small for Gestational Age at Term Fetuses

**DOI:** 10.3390/jcm11133852

**Published:** 2022-07-03

**Authors:** Anne Karge, Silvia M. Lobmaier, Bernhard Haller, Bettina Kuschel, Javier U. Ortiz

**Affiliations:** 1 Division of Obstetrics and Perinatal Medicine, Department of Obstetrics and Gynecology, University Hospital Rechts der Isar, Technical University of Munich, 81675 Munich, Germany; anne.karge@mri.tum.de (A.K.); silvia.lobmaier@mri.tum.de (S.M.L.); bettina.kuschel@mri.tum.de (B.K.); 2 Institute of AI Medical Informatics in Medicine, University Hospital Rechts der Isar, Technical University of Munich, 81675 Munich, Germany; bernhard.haller@mri.tum.de

**Keywords:** cerebroplacental ratio, uterine artery Doppler, small for gestational age, adverse perinatal outcome

## Abstract

The aim of this study was to evaluate the association between cerebroplacental ratio (CPR), mean uterine artery (mUtA) Doppler and adverse perinatal outcome (APO) and their predictive performance in fetuses with birth weight (BW) <3rd centile (very small for gestational age, VSGA) in comparison with fetuses with BW 3rd–10th centile (small for gestational age, SGA). This was a retrospective cohort study including singleton pregnancies delivered at term (37 + 0–41 + 6) in a single tertiary referral center over a six-year period. APO was defined as a composite of cesarean section for intrapartum fetal compromise (IFC), umbilical artery pH < 7.20, and admission to the neonatal intensive care unit for >24 h. The characteristics of the study population according to BW (VSGA and SGA) as well as the presence of composite APO were assessed. The prognostic performance of CPR and mUtA-PI was evaluated using receiver operating characteristic (ROC) analysis. In total, 203 pregnancies were included. Of these, 55 (27%) had CPR <10th centile, 25 (12%) mUtA-PI >95th centile, 65 (32%) VSGA fetuses, and 93 (46%) composite APO. VSGA showed a non-significantly higher rate of composite APO in comparison to SGA (52% vs. 43%; *p* = 0.202). The composite APO rate was significantly higher in SGA with CPR <10th centile (36% vs. 13%; *p* = 0.001), while in VSGA with CPR <10th centile was not (38% vs. 35%; *p* = 0.818). The composite APO rate was non-significantly higher both in VSGA (26% vs. 10%; *p* = 0.081) and SGA (14% vs. 6%; *p* = 0.742) with mUtA-PI >95th centile. The ROC analysis showed a significantly predictive value of CPR for composite APO in SGA only (AUC 0.612; *p* = 0.025). A low CPR was associated with composite APO in SGA fetuses. VSGA fetuses were more frequently affected by composite APO regardless of Doppler values. The predictive performance of CPR and uterine artery Doppler was poor.

## 1. Introduction

Fetuses with a birthweight (BW) <10th centile are classified as small for gestational age (SGA) [1]. Among them, there is a subset of fetuses with different clinical behaviors. Compared to merely constitutional SGA, growth-restricted fetuses are more often affected by adverse perinatal outcomes (APOs) including operative delivery, low Apgar scores, low umbilical artery (UA) pH, neonatal intensive care unit (NICU) admissions, hypotension, poor thermoregulation, hypoglycemia, intrauterine fetal death, and neonatal death [2,3,4]. Maternal underperfusion of the placenta is a common finding in fetal growth restriction (FGR) and could explain the differences in the pathophysiology of constitutional SGA and FGR [5]. Since a low cerebroplacental ratio (CPR) reflects a redistribution of cardiac output towards the brain due to placental dysfunction, it may identify fetuses at higher risk of APO [6,7]. In addition, an elevated mean uterine artery pulsatility index (mUtA-PI) is associated with a higher risk of cesarean section for fetal distress as well as APO [8,9]. However, its main impact is still considered as a predictor for pre-eclampsia [10,11,12].

Very small for gestational age (VSGA) fetuses with BW <3rd centile represent a particular subgroup showing a higher risk of APO [13,14]. Doppler performance in these fetuses has rarely been evaluated. Although there is a large variety of literature focusing on CPR and mUtA-PI, their meaning for APO prediction in VSGA at term remains unclear. Our aim was therefore to evaluate the association between CPR, mUtA-PI, and APO and their predictive performance in VSGA compared to SGA.

## 2. Materials and Methods

### 2.1. Participants and Protocol

This was retrospective cohort study performed at the University Hospital rechts der Isar in January 2012 and December 2017. The inclusion criteria were singleton pregnancies, cephalic presentation, mUtA-PI measurements from 32 + 0 weeks of gestation onwards, CPR measurements within one week of delivery, and delivery of an alive newborn between 37 + 0 and 41 + 6 weeks of gestation with a birth weight <10th centile. VSGA and SGA were defined as a BW <3rd centile and a BW in the 3rd–9th centile, respectively [15]. Fetuses with anatomical or chromosomal abnormalities, pregnancies with elective CS and women with abnormal labor progression (protraction or arrest at the first or second stage of labor) were excluded.

Gestational age (GA) was calculated based on measurements of the crown–rump length in the first trimester. Either a Voluson E8 (GE Medical Systems, Solingen, NRW, Germany) or a Voluson E10 (GE Medical Systems, Solingen, NRW, Germany) with 4- to 6-MHz curvilinear abdominal transducer was used. Fetal biometry was performed by measuring the biparietal diameter, head circumference, abdominal circumference, and femur length. The EFW and its centiles were calculated [15,16]. Doppler assessment of UtA, UA, and MCA was routinely performed according to our protocol for pregnancies at ≥32 + 0 weeks of gestation by doctors with at least two years’ experience in obstetric ultrasound, adhering to standardized recommendations [17]. mUtA-PI was obtained by averaging the PI values from the right and left uterine arteries. An mUtA-PI >95th centile was considered abnormal. The CPR was calculated by using MCA-PI divided by UA-PI. A cutoff <10th centile was considered abnormal according to its better performance regarding APO as compared to CPR <5th centile or CPR < 1 [18].

Composite APO was defined as the occurrence of at least one of the following parameters: CS for intrapartum fetal compromise (IFC), umbilical artery pH < 7.20, or admission to the neonatal intensive care unit (NICU) for >24 h. IFC was defined as a persistent pathological CTG pattern or the combination of a pathological CTG pattern and a fetal scalp pH < 7.20. The CTG pattern was evaluated according to the International Federation of Gynecology and Obstetrics (FIGO) criteria [19]. A pathological CTG pattern was initially managed conservatively (left lateral decubitus position, intravenous tocolysis). Fetal scalp blood sampling was indicated at the discretion of the attending obstetrician. If fetal scalp blood sampling was not possible due to cervical conditions and if the CTG pattern persisted in being pathological for 10 min after starting conservative management, CS was indicated.

### 2.2. Data Collected

The following parameters were obtained and analyzed: maternal age, body mass index (BMI), parity, ethnicity, nicotine use, pre-existing conditions, GA at ultrasound, amniotic fluid index, mUtA-PI, UA-PI, MCA-PI, CPR, CPR centiles [20,21], induction of labor, CTG assessment, fetal scalp pH, mode of delivery, GA at delivery, sex, BW, BW centile, UA pH, and Apgar score at 5 min.

### 2.3. Statistical Analysis

We used IBM SPSS Statistics for Windows, version 26 (IBM Corp., Armonk, NY, USA) for analysis. Quantitative data were shown as median and interquartile range. Categorical data were presented as absolute and relative frequencies. Differences in the distributions of quantitative variables between groups were tested using the Mann–Whitney U test. Categorical data were compared between groups using Pearson’s chi-square test or Fisher’s exact test. All statistical tests were conducted two-sided and a *p*-value < 0.05 was considered statistically significant. 

Characteristics of the study population according to BW (VSGA/SGA) as well as the presence of composite APO were analyzed. Moreover, univariate logistic regression analyses stratified by BW (VSGA/SGA) were carried out using maternal characteristics (age, BMI, parity, ethnicity, nicotine use, pre-existing conditions) and ultrasound parameters (EFW, EFW centile, CPR, CPR centile, CPR <10th centile, mUtA-PI, mUtA-PI centile, mUtA-PI >95th centile, sex) as independent variables with composite APO as a binary outcome. Statistically significant variables in the univariate analysis were considered in a multivariable logistic regression model. Finally, values of CPR and mUtA-PI, both alone and combined (multivariable logistic regression), for predicting composite APO were evaluated using receiver operating characteristics (ROC) analysis stratified by BW (VSGA/SGA).

## 3. Results

### 3.1. Baseline Characteristics and Perinatal Outcome

A total of 203 pregnancies were enrolled. Fourteen women had pre-existing conditions (four cases of systemic lupus erythematosus, four cases of essential hypertension, three cases of preeclampsia, two cases of thrombophilia, one case of type 1 diabetes). In the whole study group, 55 (27%) fetuses showed a CPR <10th centile, 25 (12%) women had a mUtA-PI >95th centile, and 10 (5%) pregnancies showed a CPR <10th centile and a mUtA-PI >95th centile. Induction of labor was performed in 131 (65%) pregnancies (93 dinoprostone vaginal insert, 38 intravaginal minprostin gel) and the most frequent indications were premature rupture of membranes ≥12 h, SGA with normal CPR from 40 + 0 weeks onwards or with low CPR from 37 + 0 weeks onward, or VSGA from 37 + 0 weeks onwards. APO included 33 (16%) cases of CS for IFC, 48 (24%) newborns with umbilical artery pH < 7.20, and 29 (14%) newborns admitted to NICU >24 h (reasons were hypoglycemia (11/29), respiratory distress (9/29), hypothermia (4/29), infection (3/29) or hyperbilirubinemia (2/29)). Composite APO occurred in 93 (46%) cases. 

Overall, 65 (32%) newborns were VSGA and 138 (68%) SGA (Table 1). There were no significant differences in maternal age, BMI, nulliparity, ethnicity, nicotine use, pre-existing conditions, GA at measurement of mUtA-PI, and CPR measurement to delivery intervals between the groups. As expected, BW and BW centiles were lower in the VSGA group. In addition, VSGA showed a lower rate of UA pH < 7.20 and a higher rate of CS for IFC, admission to NICU >24 h, and composite APO in comparison to SGA.

Regarding perinatal outcome within the groups (Table 2), VSGA pregnancies with composite APO showed a significantly higher proportion of nulliparity and induction of labor as well as a significantly lower GA at delivery and BW. SGA pregnancies with composite APO showed a significantly lower BMI, a lower proportion of Caucasian women, and a higher proportion of male fetuses.

### 3.2. Association between Doppler and Composite APO

VSGA fetuses with a CPR <10th centile or mUtA-PI >95th centile or CPR <10th centile and mUtA-PI >95th centile showed a non-significantly higher rate of composite APO (Table 2).

SGA fetuses with a CPR <10th centile had a significantly higher rate of composite APO (Table 2). In addition, SGA fetuses with composite APO showed a significantly lower CPR and CPR centile. On the contrary, mUtA-PI >95th centile or CPR <10th centile and mUtA-PI > 95th centile had a non-significantly higher rate of composite APO.

### 3.3. Logistic Regression Model

In the VSGA group, univariate logistic regression identified a significant association between one variable (nulliparity) and composite APO (Table 3). Nulliparity increased the odds of composite APO by about 13 times.

In the SGA group, univariate logistic regression identified a significant association between three variables (BMI, CPR <10th centile, male) and composite APO. However, multivariable logistic regression showed that only CPR <10th centile and male fetuses were independent predictors of composite APO (Table 3); they increased the odds of composite APO by about threefold and twofold, respectively.

### 3.4. Prognostic Value for Composite APO

In the VSGA group, a ROC analysis revealed no relevant prognostic value of CPR or mUtA-PI for composite APO, but a significant prognostic value for nulliparity (Table 4). The combined model of CPR and mUtA-PI did not improve prediction compared to mUtA-PI alone, while the combined model including nulliparity did.

In the SGA group, the ROC analysis showed a significant prognostic value of CPR for composite APO, while for mUtA-PI or male fetal sex it did not (Table 4). Both the combined model of CPR and mUtA-PI and the combined models including fetal sex (male) did not improve the prediction substantially compared to CPR alone.

## 4. Discussion

This study showed a significant association between CPR and the occurrence of composite APO in SGA fetuses. VSGA fetuses were more frequently affected by composite APO regardless of CPR value. Furthermore, uterine artery Doppler was not significantly associated with composite APO in either SGA and in VSGA fetuses. The prognostic value for composite APO of CPR and/or mUtA-PI was poor.

In the last decade, the difference between fetal growth and fetal size has been highlighted. It is important to distinguish between constitutionally small fetuses and fetuses with signs of placental insufficiency, as the latter are often affected by APO such as IFC or stillbirth, whereas the former are not [13,22,23,24]. Measurements of CPR are a part of the surveillance for SGA fetuses. Recent data suggest that pregnancies with SGA fetuses and normal Doppler findings can be prolonged safely [13]. Conversely, CPR abnormalities are associated with APO. Likewise, our data showed a significant association between low CPR and composite APO in the cohort of SGA fetuses. Previous studies have reported a poor performance of CPR as an APO predictor except for perinatal death [25,26,27]. Furthermore, Di Mascio et al. recently reported a poor performance of CPR as well as of UCA (umbilicocerebral ratio) in late-onset FGR as outcome predictors [28]. Accordingly, our analysis confirmed a low predictive performance of CPR in the SGA group. Serum parameters reflecting placental insufficiency, such as sFlt-1/PIGF, might help detect cases of SGA with a higher risk of APO, but their meaning for APO prediction in high-risk cohorts is still controversial [29,30,31,32,33].

We found that CPR values were not associated with composite APO in VSGA fetuses. This is in-line with recent data suggesting that at term EFW <3rd centile is a better APO predictor than low CPR or pathological uterine Doppler in growth restricted fetuses [13]. Furthermore, in fetuses with normal Doppler parameters, those with EFW <3rd centile showed a higher rate of CS for fetal distress and longer neonatal hospitalization compared to those with EFW >3rd centile and BW >10th centile [14]. In addition, a prospective study including a large cohort pointed out that SGA fetuses with low abdominal circumference have a particularly higher risk of being affected by APO, indicating the importance of fetal size itself in detecting FGR situations [33]. Therefore, a BW <3rd centile reflects placental insufficiency in many cases and an EFW <3rd centile should be handled as an FGR independently of the fetal Doppler status as suggested by the Delphi consensus [12]. Nevertheless, it is important to be aware of the inaccuracy of EFW as measured by ultrasound and the matching of BW. Cohort studies on EFW reported a high intra- and interobserver variability and a detection rate of less than 50% of SGA newborns [34,35]. 

In our cohort, mUtA-PI was not significantly associated with composite APO, although there was a trend in the VSGA group. However, we acknowledge that the subgroup of pregnancies with pathological uterine Doppler was small. This result is controversial, as a recently published meta-analysis implicates a similar performance of uterine artery Doppler compared to CPR for the prediction of APO in late-onset SGA fetuses [5]. In particular, the association of perinatal death or stillbirth is noteworthy not only in SGA, but also in AGA fetuses [5,36]. Still, the current suggested definition of late FGR does not include mUtA-PI [12]. In contrast to CPR, uterine Doppler reflects the maternal site of the placenta, and its elevation might be caused by an insufficient invasion of the trophoblast in the first trimester [37,38]. De novo elevations in the third trimester are more likely explained by a maternal cardiovascular maladaptation [39]. The meaning of uterine artery Doppler in VSGA pregnancies remains unclear.

Our findings showed that nulliparity was a significant predictor of APO in the VSGA cohort. An explanation for this finding might be the longer duration of the second stage of labor in nulliparous women, which is associated with APO [40]. As VSGA are already affected by a reduced placental capacity, they are more likely to experience APO—especially when the placental oxygenation is further decreased by contractions [41].

Our study was limited by its retrospective design. Furthermore, our institution is a tertiary referral center which can lead to selection bias. Moreover, since ultrasound and Doppler examinations were performed by different operators, the internal validity may be limited. Finally, as we routinely recommend induction of labor ≥37 weeks gestation for FGR fetuses (VSGA irrespective of Doppler values, SGA with abnormal Doppler), but prolongation for SGA fetuses with normal Doppler parameters, this might distort the results of this study. However, this is a common problem affecting all studies investigating APO prediction in SGA cohorts.

## 5. Conclusions

In conclusion, low CPR was associated with composite APO in SGA fetuses, but not in VSGA fetuses. VSGA is a condition that requires particularly careful monitoring regardless of the Doppler parameters. Further clinical research is warranted to clarify the role of cerebral blood flow redistribution and uterine artery Doppler in the prediction of APO as well as in the pathophysiology of the underlying placental insufficiency in VSGA fetuses.

## Figures and Tables

**Table 1:** Characteristics and outcomes of the study population according to birth weight.

	VSGA (*n* = 65)	SGA (*n* = 138)	*p*
Maternal age (years)	30.6 (7.1)	32 (6.4)	0.270
BMI (kg/m^2^)	21.6 (3.4)	21.7 (4.3)	0.947
Nulliparity	49 (75)	91 (66)	0.175
Caucasian	61 (96)	133 (96)	0.471
Smoking	6 (9)	6 (4)	0.205
Pre-existing conditions	4 (6)	10 (7)	1.000
EFW (gram)	2699 (1530)	2890 (484)	0.011 *
EFW centile	5 (10)	9 (41)	0.014 *
CPR	1.53 (0.62)	1.61 (0.57)	0.079
CPR centile	24 (42)	33 (42)	0.040 *
CPR < 10th centile	24 (37)	31 (23)	0.031 *
mUtA-PI	0.69 (0.33)	0.68 (0.23)	0.998
mUtA-PI centile	50 (71)	46 (59)	0.709
mUtA-PI > 95th centile	12 (19)	13 (9)	0.067
Measurement of mUtA-PI (weeks)	37.3 (3.8)	36.5 (5.4)	0.210
Amniotic fluid index (cm)	9.9 (6.2)	11.5 (5.4)	0.114
Induction of labor	43 (66)	88 (64)	0.740
GA at delivery (weeks)	39.4 (2.2)	40.1 (1.8)	0.019 *
CPR to delivery interval (days)	2 (5)	2 (4)	0.362
Delivery at ≥40 weeks	23 (35)	75 (54)	0.012 *
Cesarean section for IFC	15 (23)	18 (13)	0.071
UA pH	7.26 (0.09)	7.27 (0.13)	0.558
UA pH < 7.20	14 (22)	34 (25)	0.628
Apgar 5 min	10 (1)	10 (1)	0.568
Apgar 5 min < 7	1 (2)	2 (1)	1.000
Male	35 (54)	83 (60)	0.396
Birthweight (g)	2725 (280)	2870 (210)	<0.001 *

Data are expressed as median (interquartile range) or *n* (%). VSGA very small for gestational age; SGA small for gestational age; BMI body mass index; EFW estimated fetal weight; CPR cerebroplacental ratio; mUtA-PI mean uterine artery pulsatility index; IFC intrapartum fetal compromise; UA umbilical artery; GA gestational age; NICU neonatal intensive care unit; APO adverse perinatal outcome. A *p*-value < 0.05 was considered as statistically significant (*).

**Table 2:** Characteristics of the entire study population according to the composite adverse perinatal outcome (APO).

	VSGA (*n* = 65)			SGA (*n* = 138)		
	Composite APO		*p*	Composite APO		*p*
	no (*n* = 31)	yes (*n* = 34)		no (*n* = 79)	yes (*n* = 59)	
Maternal age (years)	30.7 (9.1)	29.8 (6.1)	0.533	31.9 (7.2)	32.1 (5.8)	0.952
BMI (kg/m^2^)	21.5 (3.7)	21.6 (4.6)	0.536	22.3 (4.5)	20.9 (4.5)	0.019 *
Nulliparity	17 (55)	32 (94)	<0.001 *	49 (62)	42 (71)	0.261
Caucasian	30 (97)	31 (91)	0.615	79 (100)	54 (92)	0.013 *
Smoking	3 (10)	3 (9)	1.000	4 (5)	2 (3)	1.000
Pre-existing conditions	0 (0)	4 (12)	0.115	5 (6)	5 (8)	0.744
EFW (gram)	2809 (460)	2568 (748)	0.176	2885 (513)	2890 (523)	0.679
EFW centile	5 (10)	5 (8)	0.642	9 (9)	10 (14)	0.833
CPR	1.49 (0.63)	1.61 (0.58)	0.834	1.62 (0.53)	1.59 (0.71)	0.025 *
CPR centile	25 (45)	18 (41)	0.773	35 (33)	32 (59)	0.039 *
CPR < 10th centile	11 (35)	13 (38)	0.818	10 (13)	21 (36)	0.001 *
mUtA-PI	0.64 (0.29)	0.73 (0.41)	0.053	0.68 (0.22)	0.67 (0.26)	0.735
mUtA-PI centile	41 (64)	66 (73)	0.054	49 (59)	46 (59)	0.919
mUtA-PI >95th centile	3 (10)	9 (26)	0.081	5 (6)	8 (14)	0.742
CPR <10th centile and mUtA-PI >95th centile	2 (6)	5 (15)	0.430	1 (1)	2 (3)	1.000
GA at measurement of UtA-PI	37.5 (4.0)	37.1 (1.9)	0.703	36.3 (5.9)	35.7 (5.1)	0.632
Amniotic fluid index (cm)	9.5 (6.3)	10.1 (7.0)	0.702	11.5 (5.9)	11.5 (5.8)	0.820
Induction of labor	16 (52)	27 (79)	0.018 *	45 (57)	43 (73)	0.054
GA at delivery (weeks)	39.8 (1.2)	39.0 (2.4)	0.035 *	40.0 (1.9)	40.1 (2.1)	0.514
CPR to delivery interval (days)	1.5 (7.0)	3.0 (5.0)	0.384	2.0 (5.0)	1.5 (3.0)	0.423
Delivery at ≥40 weeks	13 (42)	10 (29)	0.292	42 (53)	33 (56)	0.747
Male	14 (45)	16 (47)	0.878	25 (32)	30 (51)	0.023 *
Birthweight (g)	2760 (185)	2640 (370)	0.048 *	2855 (200)	2917 (220)	0.101
Birthweight centile	2 (0)	2 (0)	0.340	7 (2)	8 (2)	0.120

Data are expressed as median (interquartile range) or *n* (%). VSGA very small for gestational age; SGA small for gestational age; BMI body mass index; EFW estimated fetal weight; CPR cerebroplacental ratio; mUtA-PI mean uterine artery pulsatility index; GA gestational age. A *p*-value < 0.05 was considered as statistically significant (*).

**Table 3:** Univariate and multivariable logistic regression of predictors of composite adverse perinatal outcome. Variables significantly associated with adverse perinatal outcome in univariate regression (*p* < 0.05) were included in the multivariable model.

	Univariate OR	95% CI	*p*	Multivariable OR	95% CI	*p*
**VSGA**						
Maternal age (years)	0.959	0.873–1.053	0.378			
BMI (kg/m^2^)	0.997	0.883–1.125	0.958			
Nulliparity	13.17	2.676–64.87	0.002 *			
Caucasian	0.344	0.034–3.499	0.368			
Nicotin use	0.903	0.168–4.846	0.905			
Pre-existing conditions	3.759	0.717–19.074	0.117			
EFW	0.999	0.998–1.001	0.250			
EFW centile	1.001	0.951–1.053	0.968			
CPR	1.115	0.358–3.468	0.851			
CPR centile	0.998	0.981–1.016	0.855			
CPR <10th centile	1.126	0.410–3.090	0.818			
mUtA-PI	6.153	0.599–63.22	0.126			
mUtA-PI centile	1.013	0.999–1.027	0.078			
mUtA-PI >95th centile	3.360	0.817–13.812	0.093			
Male	1.079	0.406–2.866	0.878			
**SGA**						
Maternal age (years)	1.010	0.948–1.076	0.762			
BMI (kg/m^2^)	0.897	0.814–0.988	0.027 *	0.918	0.834–1.010	0.078
Nulliparity	1.513	0.733–3.119	0.262			
Caucasian	0.171	0.019–1.571	0.119			
Nicotin use	0.658	0.116–3.718	0.636			
Pre-existing conditions	1.408	0.545–3.641	0.480			
EFW	1.000	0.999–1.001	0.688			
EFW percentile	1.004	0.968–1.042	0.819			
CPR	0.445	0.190–1.039	0.061			
CPR percentile	0.992	0.980–1.004	0.182			
CPR < 10th percentile	3.813	1.629–8.928	0.002 *	2.804	1.133–6.944	0.026 *
mUtA-PI	1.012	0.156–6.556	0.990			
mUtA-PI percentile	0.999	0.988–1.010	0.877			
mUtA-PI >95th percentile	0.822	0.255–2.653	0.743			
Male	2.234	1.113–4.485	0.024 *	2.227	1.035–4.792	0.040 *

OR odds ratio; CI confidence interval; VSGA very small for gestational age; SGA small for gestational age; BMI body mass index; EFW estimated fetal weight; CPR cerebroplacental ratio; mUtA-PI mean uterine artery pulsatility index. A *p*-value < 0.05 was considered as statistically significant (*).

**Table 4:** Receiver operating characteristics analysis of Doppler parameters alone and combined to predict composite adverse perinatal outcome stratified by birthweight.

	AUC	95% CI	*p*
**VSGA**			
CPR	0.515	0.372–0.658	0.834
mUtA-PI	0.639	0.504–0.775	0.054
Nulliparity	0.696	0.565–0.828	0.007 *
CPR + mUtA-PI	0.641	0.506–0.777	0.051
CPR + nulliparity	0.706	0.576–0.836	0.004 *
mUtA-PI + nulliparity	0.814	0.710–0.917	<0.001 *
CPR + mUtA-PI + nulliparity	0.809	0.705–0.914	<0.001 *
**SGA**			
CPR	0.612	0.512–0.712	0.025 *
mUtA-PI	0.517	0.419–0.615	0.735
Male	0.596	0.500–0.692	0.054
CPR + mUtA-PI	0.611	0.511–0.711	0.026 *
CPR + male	0.647	0.550–0.743	0.003 *
mUtA-PI + male	0.590	0.492–0.688	0.071
CPR + mUtA-PI + male	0.649	0.552–0.745	0.003 *

AUC area under the curve; CI confidence interval; VSGA very small for gestational age; SGA small for gestational age; CPR cerebroplacental ratio; mUtA-PI mean uterine artery pulsatility index. A *p*-value < 0.05 was considered as statistically significant (*).

## Data Availability

Not applicable.

## Abbreviations

APO	Adverse perinatal outcome
BMI	Body mass index
BW	Birth weight
CI	Confidence interval
CPR	Cerebroplacental ratio
EFW	Estimated fetal weight
FGR	Fetal growth restriction
FIGO	Federation of Gynecology and Obstetrics
GA	Gestational age
IFC	Intrapartum fetal compromise
mUtA	Mean uterine artery
NICU	Neonatal intensive care unit
PI	Pulsatility index
ROC	Receiver operating characteristic
SGA	Small for gestational age
UA	Umbilical artery
VSGA	Very small for gestational age
